# Hermaphrodism, from a Medico-Legal Point of View

**Published:** 1875-09

**Authors:** 


### Translations.

### HERMAPHRODISM,

### FROM A MEDICO-LEGAL POINT OF VIEW.

*Translated from the French of Basile Poppesco,*

By E. WARREN SAWYER, M.D.,
Lecturer on Obstetrics, Rush Medical College, Chicago.

### INTRODUCTION.

The vices of conformation of the genital organs had from all time attracted the notice of observers; but the absence of positive notions of the normal anatomy and pathology, to the middle of the eighteenth century, had permitted the ranging of these facts, which were much more in the domain of the marvellous and unknown, in a special category, rather than in the true domain of science.

It was only at the end of the last century, and the beginning of this, that numerous works appeared which partly elucidated the subject and enabled us to penetrate the infinite modifications of its mechanism. However, it was necessary to wait for the recent discoveries of modern anatomy, and the labors in embryology, in order to affirm that the question of hermaphrodism was nearly solved.

The medico-legal questions, which are attached to this arrest of development, can be treated now with an indisputable competence, before the tribunals, because the medical jurist has as a base for his argumentation, and foundation for his declarations, the entire group of phenomena amassed from embryology and experimentation.

Prof. Tardieu, in his lectures, and in his works, is one of those who have contributed most to elucidate this subject, and to show all its practical bearings from a medico-legal point of view. We have thought that, in the present state of science, a summary of the principal questions which belong to hermaphrodism would not be deprived of all interest; but in penetrating the difficulty of the subject, we feel constrained to beseech of our judges all the kindness possible.

HISTORY. VARIETIES, CLINICAL AND SYMPTOMATIC.

The origin of this word goes back to the remotest antiquity and seems to be attached to the fable of Hermaphrodite, son of Mercury (*Ερμῆς*) and of Venus (*Α'φροδι'τη*), who remaining insensible to the charms of the nymph Salmacis, she obtained from the gods the decree that her body might be united to his.

Later, the possibility that an individual could at once possess the attributes of both sexes was fully admitted, and already the Fathers of the church, Tertullien among others, are seen violently opposed to them; and if the Athenians throw into the sea the reputed hermaphrodites, the Romans are seen not to fail in employing the same kind of execution by precipitating them into the Tiber.

In the middle age, ignorance and passion still follow these unhappy individuals, disinherited from nature; and we will be able to recall, in this connection, numerous decrees of the provincial parliaments.

In the rigorous acceptation of the word, one will understand an hermaphrodite to be an individual who is capable of reproducing one of his species without the concurrence of another. This will be, in the animal kingdom, an exceptional peculiarity which will enable us to establish a complete similarity with what commonly passes in the greatest number of vegetable families; one class only in the vegetable kingdom, the *Diœcia*, constituting an exception.

In the animal kingdom, as one descends the scale there appear animal hermaphrodites; thus, for example, among the mollusks, and the zoöphytes; but here still a distinction should be established; although possessing male and female organs, among some species, the concha univalvata, for example, the animal requires, in order to be fecundated, the intervention of another individual of his kind; in this case, the animal is at once fecundating and being fecundated.

On the contrary, take the concha bivalvata, and one will admire at once with what care and harmony the laws of nature have been established, in order that the fecundation of these creatures, their unceasing repetition, may not be interrupted; for here the animal has a fixed domicile, permanent; it is impossible for him to leave his rock; in the act of fecundation he has no need of the help of another individual of his kind; he is at once fecundating and being fecundated.

I will not extend farther these considerations of comparative anatomy; I only wish to show the insensible gradation that one meets with in the attentive observation of nature; at the same time to show what an immense distance separates the bisexuality of the inferior animals from this that has always been termed in man *hermaphrodism* or *androgynia*.

As M. Tardieu has remarked,* "the name of hermaphrodite, under which one usually designates these individuals, is the worst that can be chosen, and gives the most false idea of their conformation." One may say that it is not important which may be the predominant sex, the one or the other ; often, even, both are too incompletely developed to be able to fill their *rôle ;* the association has never, however, been perfect; it is scarcely so in two or three recent observations where the simultaneous presence of the essential and accessory organs of the male and female sex have been established ; I will make, however, an exception for an observation reported by M. Doumic, † due to M. le Dr. Heppner, of St. Petersburg, also for two observations which are found at the end of this thesis ; one taken from the journal *Lo Sperimentale* of Florence (1874), the other from the *Lyon Médical* same year.

Haller, ‡ relying on the considerations of structure, had already said that the union of the two sexes was absolutely irreconcilable with the disposition of the pelvis ; and later, Meckel, and Serves especially, really corroborate this opinion in taking for the base of their refutation the law, called by the latter, the law of balancing of the organs.

In the last century, we see already the authors divided into two parties, regarding the sex of the presumed hermaphrodites: one, with Osiander, thought that they are nearly all males, while Parsons § and Hill, on the contrary, supposed them nearly all females.

To-day, the almost unanimous opinion is, that in the great majority of cases, that are wrongly called hermaphrodites, they are individuals of the male sex in

* Question médico-légale de l'indentité dans ses rapports avec les vices de conformation des organes sexuels, 1874.

† Doumic, Gazette Med., Paris, 1872.

‡ Comm. Soc. R. Gott., t. 1, pl. 1.

§ Parsons' Medical and Critical Inquiry into the Nature of Hermaphrodites. London, 1741.

whom the testicles have descended and are atrophied ; or rather, on the contrary, remain included within the cavity of the abdomen ; in whom a penis is recognized, having an incomplete development ; a urethra more or less opened at the base of the penis ; finally, a failure to unite of the two lateral parts which had as their special destination, on account of their approaching each other and their intimate adherence, the forming of the superficial coverings of the pouches, the scrotum, strictly speaking. In a word, the arrest of development which constitutes, strictly, hermaphrodism, consists essentially in two special alterations of the external and internal genital organs, which have been designated under the name of hypospadias and cryptorchidia ; as M. Tardieu does not hesitate to say, "that one will not be in doubt that these are malformed men, but men." The same opinion is equally supported by M. Legrand du Saulle in his Treatise of Legal Medicine (1874).

Nevertheless, though the arrest of development in the male sex constitutes the greatest part of the facts in science under the name of hermaphrodism, we will describe, after the authors, the three most important varieties ; we believe this division necessary, not only because we will be enabled better to group the facts, but because it accords with the observation and with the minute description given by Is. Geoffroy Saint Hiliare, Dutrochet and Marc.*

### I. APPARENT HERMAPHRODISM IN THE MALE SEX.

Sometimes the arrest of development involves only the generative organs, sometimes there are phenomena worthy of being spoken of which belong to the general configuration of the individual. We will first describe the former, or phenomena localized in the generative sphere, properly speaking.

The penis offers, usually, an incomplete development ;

* Dict. en 30 ; Art. Hermaphrodisme.

its length is more or less reduced; it is formed by the union, the fusion of the two corpora cavernosa; the glans is often malformed; moreover, if it exists it remains uncovered, in consequence of the absence, or the want in length, of the prepuce. One will readily admit that if the volume of this organ is very slight, not exceeding in length two-thirds of an inch or an inch, it may sometimes be difficult to distinguish it from the clitoris, if besides there are added to this other vices of conformation. This penis is capable of an erection, and the intromission of this organ will depend directly upon the length which it can acquire at the moment of venereal spasm.

Below this penis, one recognizes a deformity also very important; we allude to that arrest of development which has received the name of hypospadias: there are a number of varieties of this; sometimes the urethra is opened at a point very nearly approached to the terminal extremity of the penis, and then it is not a true hermaphrodism; sometimes, on the contrary, it is only towards the middle of the penis that a canal is observed which opens directly into the bladder; finally, the most frequent variety is that in which an opening is found quite at the base of the penis, an orifice which is just beneath the pubis, in an infundibuliform cavity which we will describe presently.

In this case, one observes also the co-existence of other arrests of development. We know that the scrotum is the result of the intimate union of the two lateral folds which unite in the median line and form a well marked raphe, which corresponds superiorly to what is known under the name of the septum of the dartos. When this union of these lateral folds does not take place, and these two external processes from the blastoderm remain separated, there will result an absence of the scrotum, and each side will have the gross appearance of the labium majorum.

These two separated parts leave on the median line a fissure, which extends from before backwards; sometimes this is very superficial, sometimes, on the contrary, it is a true hollow cavity, more or less funnel shaped. This cavity does not extend very far forward, the finger is soon arrested, and at the anterior and superior part one can recognize the abnormal opening of the urethra. If one wishes to carry the examination farther, the catheter may be introduced nearly into the bladder and the finger into this abnormal cavity, when the presence of the prostate, and even the vesiculæ seminales, will be established; most frequently, however, it will be nearly impossible to arrive at such precision in the diagnosis, the cavity being too shallow and the rectal touch giving but very uncertain information.

But a fact which leads still more to the confounding of the external conformation of these subjects with the special conformation of the female sex, is the absence of the testicles, or at least their permanent inclusion within the abdominal cavity. One will understand how necessary it is in these cases to practice a minute palpation; because the absence or presence of these organs goes to indicate more exactly, for the medical jurist, and for the expert, the course which he will follow in the decision which is necessary for the proper ranking of this individual in the civil state; or the motives which he can allege in favor of a rupture of the marriage.

Not only the lateral pouches which represent the scrotum ought to be examined, but the inguinal ring and the entire inguinal tract should be explored; because it is not exceptional to find, in those cases of presumed hermaphrodites, the testicles retained within the inguinal canal, from which their descent does not take place till the epoch of puberty. Finally, one should recall the interesting case of Alexina B——, reported by M. le Prof. Tardieu, in which the testicles were retained within the inguinal canal, and were the seat of a most violent congestion at the time of puberty, which was, for the physician, the first indication of a vice in conformation which was presented by this presumed young girl.

Moreover, in the variety of hermaphrodism with which we are occupied at present, the variety most frequently observed, one can say that the principal characteristics are comprised in the following observations: diminution in the size of the penis; hypospadias, generally well marked; absence of union of the two lateral parts which normally go to form the scrotum; and finally, cryptorchidie.

To these local signs of hermaphrodism, may be added a series of phenomena which belong to the general conformation of the individual; and often, too, not only are the external physical characters similar to the female exterior, but the real character, the habits, the passions, conform to the female character rather than to the male.

The stature is generally medium; the rotundity of body (l'embonpoint) is considerable; the skin is smooth and fine, deprived of hair upon the face as well as upon the trunk; the muscular system is slightly developed, or rather it is marked by a full adipose development; the muscular projections and the bony ridges are effaced; the extremities, in a word, like the face, have the appearance of the feminine type. The voice even has a high pitch, and is feeble. The pelvis is sufficiently developed, and the ilia project outwards; there is even an abnormal turning outwards of the ischio-pubic branches. The breasts are of considerable size. These individuals prefer sedentary occupations; they like garments of brilliant colors; are excessively coquettish and adore perfumes; an oriental extravagance reigns in their apartments (obs. iv); they are also passionately fond of needle-work, etc.; in a word, everything in the outward character contributes in keeping up the error as to the real sex of these individuals.

At the end of this essay, several observations can be seen which relate clearly to this form of hermaphrodism, therefore I will not stop to detail those examples, which are classical to-day, of Marie Marguerite, of Dreux, related by Worbes, which is found in the journal of Hufeland. To this same variety belongs equally the case of Justine, A. J., which was decided by the civil tribunal of Alais (1869), then by the courts of Montpelier (1872), and of Nimes, after the first decision had been reversed by the Court of Cassation (1869); in the last place it was finally brought back before the civil tribunal of Alais (1873): the court then declared the individual "absolutely nothing, a nonentity, and annulled the marriage inscribed upon the registers of the civil state, and, consequently, the ante-nuptial pledge, which governed the agreements of the parties; and ordered that mention of the present decision be made on the margin of the act of celebration of the aforesaid marriage, thus annulled by the officer of the civil state of the said commonwealth of Alais."

### II. APPARENT HERMAPHRODISM IN THE FEMALE SEX.

It is much more rare, as we have already said, to encounter in the female a disposition of the sexual organs such as could lead one in error as to their true sex, and consequently, such as would lead one to suppose that he had to do with an individual of the male sex. Nevertheless, several such cases exist in science, and these are, in summary, the peculiarities presented to the observer. The vagina is reduced to a simple funnel-shaped hollow cavity; it is imperforated or narrowed by bridles, which are so numerous that it is impossible to find the neck of the uterus by the touch; one cannot reach this organ than by a methodical palpation and by the simultaneous employment of the catheter and the rectal touch. The clitoris may be as voluminous as in the case of the hypospadias which we have related above; finally, singularly coincident, the meatus urinarius, in consequence of an unusual elongation of the urethra, may be found just beneath the clitoris, which fact increases the chance of error, when only a simple superficial examination is made. The labia majora, in these cases, are but slightly pronounced; often the nymphæ are scarcely marked, in consequence of an arrest of development in all the parts. Finally, a last fact, which is prominent in some instances, and which still more singularly obscures the diagnosis, is the presence of the ovaries in the substance of the labia majora, in consequence of an inguinal hernia. It is then that only a most carefully conducted examination will enable us to differentiate the testicles from the ovaries (*Estopie ovarienne.* Loumaigne, thèse inaugurale 1869). But, we repeat it, this variety is rare—really exceptional. In the great majority of the cases, it will be easy enough to establish this variety of hermaphrodism, which consists really and simply in an arrest of development, in a default of communication between the external and internal genital parts. It is true that, in these cases, *the ovaries, the sign par excellence of the female sex*, exist—sometimes displaced, sometimes in their normal situation—and indicate their presence not by the menses, which cannot appear, but by various periodical troubles, symptoms of congestion, increased secretions, hæmorrhages, etc., which resemble really the regular menstrual evolution.

Add to these local signs the entire group of external physical characteristics, and intellectual manifestations, which are really more conformed to the male sex than to the female, and it will be seen that it is with reason that the writers of the last century, and the beginning of this, have insisted with so much detail upon these analogous facts. The general complexion of these persons is much more masculine ; the stature is increased, the body is lank, the limbs powerful ; the muscular projections and the bony ridges are well pronounced ; the hair is short, the hair follicles are developed and the surface of the body is covered with hair ; the breasts are slightly developed ; the depth of the pelvis is increased, and the menstruation is more or less completely wanting. Add to all this, the aptitude for a series of manly tastes, a preference for the occupations which require force and vigor, a deep voice, and you will have under your eyes, in a few words, those creatures which the Romans designated, with just reason, under the name of *virago.* One feature alone which led to error in several of the cases cited was, that the clitoris was so long that it was capable of copulation; but, as M. Tardieu has said,* "this fact alone does not change the conditions upon which are founded the distinction of the sexes."

Marie-Madeleine Lefort, said Béclard,† seemed to belong to the male sex, if one considered the size of the trunk and the extremities, the shoulders and the pelvis, the size of the larynx, the tone of the voice, the development of the beard, and the urethra prolonged beyond the symphysis pubis. But she possessed at the same time the essential and constitutive organs of woman, uterus and vagina. Below, and posteriorly to the peniform clitoris, was a fissure or vulva bordered by two narrow, short labia, which were united by a thick and dense membrane. At the root of the clitoris, this membrane was perforated with a round opening which gave passage to the urine and the menstrual discharge, and would have been sufficient, without doubt, to have made the vagina accessible if an incision had been made, between these two labia, from the base of the clitoris nearly to the posterior commissure. Since this instance, analogous cases have been reported by MM. Bouillaud and Manec, Briquet, Richard, Gallard, Lefort.‡

Marc, in his article of the Dict. en 30, reports that a uterus fallen out of the vagina has sometimes led the inattentive and ignorant surgeons in error, who believed it to be a veritable penis. Such was the mistake of the surgeons and the chief magistrates of Toulouse respecting a woman whom they declared an hermaphrodite, in 1693, and ordered her to wear the clothing of males.

* Tardieu, loc. cit.

† Bulletin de la Société de la Faculté de Médicine, 1815.

‡ Lefort, Thèse d'Agrégation, 1863. Des vices de Conformation de l'utérus et du Vagin.

This woman coming to Paris, was subjected to an examination by able men, Saviard alone recognizing the error, reduced the prolapsed uterus, and gave to this woman her true sex.

Everard Home (Philos. Trans., 1799,) cites a similar case.

It seems to me sufficient to prevent falling into a like error, to know that it may be committed.

(To be continued.)

